# Metformin improves IK_Ca_-mediated endothelial dilative dysfunction of arteriole in diabetic rats

**DOI:** 10.1186/1479-5876-10-S2-A64

**Published:** 2012-10-17

**Authors:** Li-Mei Zhao, Yong Yang, Yan Wang, Xiu-Ling Deng

**Affiliations:** 1 Department of Physiology and Pathophysiology, School of Medicine, Xi’an Jiaotong University, 76 West Yanta Road, Xi’an, 710061, Shanxi, China

## Background

Activation of intermediate conductance Ca^2+^-activated K^+^ channel (IK_Ca_) in endothelial cells has been shown to contribute to vasodilation, especially in small vessels. The aim of this study is to observe the effect of metformin on endothelial dilative dysfunction in diabetic rats and investigate whether the alteration of IK_Ca_ are involved in the underlying mechanism.

## Methods

Diabetic rat model was induced by a single intraperitoneal injection of 30 mg/kg STZ after high fat and glucose diet for 8 weeks. Animals whose blood glucose > 11.1 mmol/L were included in diabetic and metformin group. Age-matched animals fed with standard chow and injected with citric acid buffer were served as control. Four weeks after STZ injection, rats in three groups were fed with normal diet for additional 8 weeks. After that, fasting blood was drawn and third-order mesenteric arteries were separated. Hemoglobin A1c (HbA1c) was measured with an automatic analyzer. The changes of Ach- and NS309 (opener of IK_Ca_ and small conductance Ca^2+^-activated K^+^ channel, SK_Ca_) -induced vasodilatation mediated by IK_Ca_ in mesentery arterioles of each group and mesentery arterioles of normal rats incubated with 200 μg/mL AGE-BSA (200 μg/mL BSA as control) for 3 hours were measured by multi-myograph system. The effect of metformin on AGE-BSA (200 μg/mL) and H_2_O_2_ (100 μmol/L) induced changes of IK_Ca_ mRNA and protein expression in cultured human umbilical vein endothelial cells (HUVECs) were detected by RT-PCR and Western blot. The level of malondialdehyde (MDA) and the activity of Cu-Zn superoxide dismutase (Cu-Zn SOD) in cellular supernatant were determined by colorimetric method.

## Results

Increased HbA1c level and reduceded endothelium-dependent dilative response mediated by IK_Ca_ in mesentery arterioles were observed in diabetic rats, and metformin treatment (300 mg/kg/day by gavage) restored the adverse condition. The vasodilatation mediated by IK_Ca_ was also impaired in 200 μg/mL AGE-BSA-incubated mesentery arterioles. AGE-BSA at 200 μg/mL concentration and H_2_O_2_ (100 μmol/L) significantly decreased the mRNA and protein expression of IK_Ca_. AGE-BSA also increased the production of MDA and inhibited Cu-Zn SOD activity in HUVECs. Metformin of 10^-6^ mol/L and 10^-7^ mol/L reversed those effects.

## Conclusion

Metformin significantly improves endothelium dilative dysfunction mediated by IK_Ca_ in diabetic rats, which is likely related to the inversion of AGEs-induced oxidation and downregulation of IK_Ca_ expression in endothelial cells.

